# Pulse Shape and Timing Dependence on the Spike-Timing Dependent Plasticity Response of Ion-Conducting Memristors as Synapses

**DOI:** 10.3389/fbioe.2016.00097

**Published:** 2016-12-26

**Authors:** Kristy A. Campbell, Kolton T. Drake, Elisa H. Barney Smith

**Affiliations:** ^1^Department of Electrical and Computer Engineering, Boise State University, Boise, ID, USA

**Keywords:** memristor, ion-conductor, non-volatile memory, ReRAM, STDP

## Abstract

Ion-conducting memristors comprised of the layered materials Ge_2_Se_3_/SnSe/Ag are promising candidates for neuromorphic computing applications. Here, the spike-timing dependent plasticity (STDP) application is demonstrated for the first time with a single memristor type operating as a synapse over a timescale of 10 orders of magnitude, from nanoseconds through seconds. This large dynamic range allows the memristors to be useful in applications that require slow biological times, as well as fast times such as needed in neuromorphic computing, thus allowing multiple functions in one design for one memristor type—a “one size fits all” approach. This work also investigated the effects of varying the spike pulse shapes on the STDP response of the memristors. These results showed that small changes in the pre- and postsynaptic pulse shape can have a significant impact on the STDP. These results may provide circuit designers with insights into how pulse shape affects the actual memristor STDP response and aid them in the design of neuromorphic circuits and systems that can take advantage of certain features in the memristor STDP response that are programmable *via* the pre- and postsynaptic pulse shapes. In addition, the energy requirement per memristor is approximated based on the pulse shape and timing responses. The energy requirement estimated per memristor operating on slower biological timescales (milliseconds to seconds) is larger (nanojoules range), as expected, than the faster (nanoseconds) operating times (~0.1 pJ in some cases). Lastly, the memristors responded in a similar manner under normal STDP conditions (pre- and post-spikes applied to opposite memristor terminals) as they did to the case where a waveform corresponding to the difference between pre- and post-spikes was applied to only one electrode, with the other electrode held at ground potential. By applying the difference signal to only one terminal, testing of the memristor in various applications can be achieved with a simplified test set-up, and thus be easier to accomplish in most laboratories.

## Introduction

Bioinspired neuromorphic computing has the potential of becoming realizable through the application of memristors (Chua, 2015) as artificial synapses (Jo et al., 2010; Chang et al., 2011; Erokhin and Fontana, 2011; Rose et al., 2011a,b; Gaba et al., 2013; Serrano-Gotarredona et al., 2013a; Subramaniam et al., 2013; Thomas, 2013; Mahalanabis et al., 2016). The spike-timing dependent plasticity (STDP) synaptic learning rule, inspired from the behavior of the biological neural system (Dayan and Abbott, 2001) and dominant in the brain, has been proposed and experimentally demonstrated with memristors acting as synapses by several groups over the past few years in many material systems, such as oxides (Yu et al., 2011; Wang et al., 2012a,b, 2016; Wu et al., 2012; Pickett et al., 2013; Mandal et al., 2014; Kim et al., 2015), chalcogenides (Li et al., 2013b; Mahalanabis et al., 2014a,b, 2016; La Barbera et al., 2015), silicon (Jo et al., 2010; Subramaniam et al., 2013), organic materials (Alibart et al., 2012; Li et al., 2013a; Cabaret et al., 2014; Luo et al., 2015), and even magnetic tunnel junctions (Krzysteczko et al., 2012). Illustrations of memristor effectiveness have also been shown in simulation and with transistor and/or complementary metal oxide semiconductor (CMOS)-based memristors (Rachmuth et al., 2011; Rose et al., 2011a,b; Cruz-Albrecht et al., 2012; Noack et al., 2015) and graphics processing units (Snider et al., 2011). The exploration of new memristor materials systems is driven by the advantage of analog, memristor-based learning implementations compared to the digital-based learning, where the analog, memristor-based learning was shown to provide an improvement of at least a factor of 10 for power and density (Rajendran et al., 2013) over digital-based learning. The larger area and power requirement for CMOS-based memristors have driven the research into novel material-based memristor STDP to find a lower power/area alternative for neuromorphic computing.

Some of the issues with previous experimental implementations of memristors in the synaptic role in the STDP application (Chang et al., 2011; Rose et al., 2011a,b; Li et al., 2013a; Subramaniam et al., 2013; Luo et al., 2015; Mahalanabis et al., 2016) include the lack of analog programmability of the memristor, high power requirements, and requirement of very specific programing spike shapes in order to effectively program the synaptic weights. Recent work, using a TaO*_x_* memristor as a synapse has demonstrated incremental switching in memristors, through the use of repetitive pulses and a pulse train with increasingly higher amplitudes. However, the pulses used in this study were limited to 100 ns pulse width (Wang et al., 2016). A similar TaO_5−_*_x_* memristor was also used (Kim et al., 2015) to demonstrate the effects of incremental pulses on the memristor resistance tuning, as well as use of pulses in the range of 100 ns to 10 µs to demonstrate the STDP response. It should be noted that the incremental resistance programing response was also demonstrated in a chalcogenide-based memristor based on a phase-change mechanism, using 30 ns pulses in a five pulse train with increasing pulse amplitude from 1 to 1.8 V to increase resistance and −0.6 to −0.8 V to decrease resistance (Li et al., 2013b).

**Figure 1:**
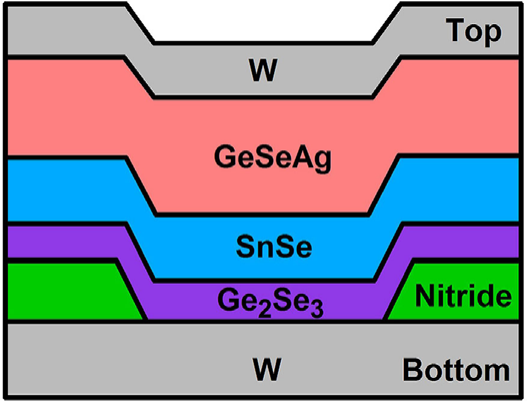
**Memristor device structure showing (from bottom to top) the bottom tungsten (W) electrode, the insulating nitride layer which defines the active layer contact area to the bottom electrode, the active layer (Ge_2_Se_3_) followed by the SnSe, GeSeAg, and top tungsten electrode layers**. The function of each layer is described in Campbell (2017).

The memristor used in this work is based on the ion-conducting self-directed-channel (SDC) memristor, which has demonstrated lifetime endurance greater than one billion cycles, operation at temperatures of 150°C without degradation, and analog programmability (Campbell, 2017). This device is comprised of chalcogenide material layers (Figure 1) (Campbell, 2008a,b, 2017). It uses a Ge_2_Se_3_ chalcogenide layer, which is activated for analog resistance tuning operation by Sn ions that migrate from an adjacent SnSe layer during the initial forming process (Campbell and Anderson, 2007; Devasia et al., 2010, 2012). A layer of ternary GeSeAg is the ion source during operation. In contrast to other Ag-based GeSe or GeS ion-conducting device types (Mitkova and Kozicki, 2002; Kozicki and Mitkova, 2006; Kamalanathan et al., 2009; Waser et al., 2009; Wang et al., 2011; Mahalanabis et al., 2014a,b; Rajabi et al., 2015; Ielmini and Waser, 2016), no photodoping or thermal annealing steps are required, simplifying the fabrication steps, and producing more consistent device operation. These differences also enable the device used in this work to withstand higher fabrication (at least 300°C) and operating temperatures (operation at 150°C is routinely performed). Additionally, this device can be integrated into a back-end-of-line (BEOL) CMOS process (Regner et al., 2009) making it compatible with CMOS architectures (Serrano-Gotarredona et al., 2013b).

The advantage of the SDC memristor used in this work, over all of the memristors described to date in the STDP application is that it is the only memristor that has been shown to be simultaneously capable of (1) operation over nanoseconds to seconds timescale in STDP; (2) analog programmability over at least four orders of magnitude of resistance; (3) operation at high temperature (150°C); (4) cycling in excess of one billion times; and (5) demonstrated incorporation into a BEOL CMOS process. The scalability is predicted to be easily below 20 nm due to the one dimensional aspect of device operation (based on success at 27 nm node of CuTe-based 16 Gb memory; Fackenthal et al., 2014).

**Figure 2:**
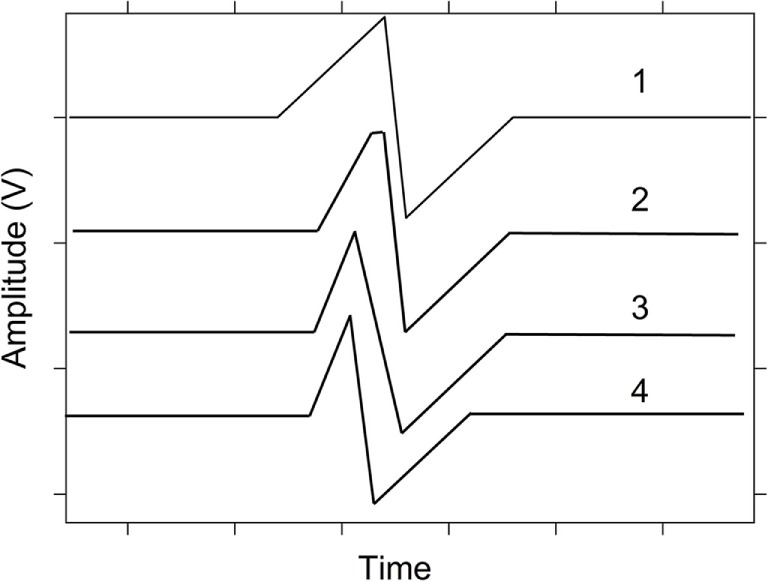
**Spike shapes 1–4 used during spike-timing dependent plasticity testing**.

The analog resistance programing capabilities of the SDC memristor used as a synapse are demonstrated in this work through the memristor’s synaptic weight change induced during the STDP experiment over the nanoseconds to seconds timescale, and the response to four different synaptic pulse shapes (Figure 2). These pulse shapes were used to explore the effects of the spike shape on the STDP response. As has been previously noted (Zhu et al., 2014; Qu et al., 2016), the ion-conducting memristive devices are logical candidates for this purpose since they have functional similarities to biological synapses in that both synapse types have a dependence on ion species to alter the synaptic strength.

In addition to the STDP pulse shape tests, the memristor response to only the resultant waveform applied to one electrode while the other electrode was held at ground potential was also measured.

## Materials and Methods

### Device Fabrication

Ion-conducting devices were fabricated with a *via* structure and top and bottom electrodes, as shown in Figure 1. The active switching layer is the 300 Å Ge_2_Se_3_ layer adjacent to the bottom electrode.

Devices were fabricated on 100 mm p-type Si wafers with the bottom electrode of 250 Å Cr/500 Å W already deposited *via* chemical vapor deposition on the wafers (purchased from Encompass Distribution Services, Tracy, CA, USA). The bottom electrode was patterned, followed by 800 Å sputtered nitride (AJA International ATC Orion 5 UHV Magnetron sputtering system). A *via* etched through this nitride layer (Oxford Plasma Etcher) defines the device contact area. *Via* sizes for this work were 4 µm in diameter and there were at least 200 devices per wafer.

Wafers were pre-sputtered with Ar^+^ to remove any oxide species from the bottom electrode, followed by *in situ* sputter deposition of all of the remaining device layers and top tungsten electrode layer, using an AJA International ATC Orion 5 UHV Magnetron sputtering system. The target layer thicknesses were (from bottom to top): Ge_2_Se_3_ (300 Å)/SnSe (800 Å)/Ge_2_Se_3_ (150 Å)/Ag (500 Å)/Ge_2_Se_3_ (100 Å)/W (400 Å). The top three layers below the tungsten top electrode, corresponding to Ge_2_Se_3_/Ag/Ge_2_Se_3_, mix during fabrication, becoming one conductive layer. Final device etching was performed with a Veeco ME1001 ion-mill. The active switching layer is the 300 Å Ge_2_Se_3_ layer deposited adjacent to the bottom electrode. The SnSe layer provides Sn ions and isolates the Ge_2_Se_3_ layer from direct contact with the Ag layer.

### Device Operation

Devices are initially in a high resistance state (megaohms to gigaohms range) following fabrication. The first programing operation applies a positive potential to the top electrode and forces Sn ions from the SnSe layer into the active Ge_2_Se_3_ layer (Campbell and Anderson, 2007; Devasia et al., 2010, 2012; Campbell, 2017). For the devices used in this work, in addition to Sn ions from the SnSe layer, Ag^+^ ions from the GeSeAg layer are also incorporated into the active layer during this first programing operation. A conductive pathway, likely comprised of multiple conductive channels (Banerjee and Chakravorty, 1999; La Barbera et al., 2015), forms due to incorporation of these ions into the active layer, resulting in a resistance drop. The resistance can be increased by application of a potential across the device that places the top electrode at a lower potential than the bottom electrode, thus forcing ions out of the conductive channel toward the top electrode. The resistance of a device is related at any time to the resultant conductivity of the active switching layer, which is in turn related to the amount of incorporated metal and organization of conductive channels within the active layer (Banerjee and Chakravorty, 1999; La Barbera et al., 2015). A description of SDC device operation and differences between the SDC device and other ion-conducting devices, such as the conductive bridged random access memory, is in Campbell (2017).

**Figure 3:**
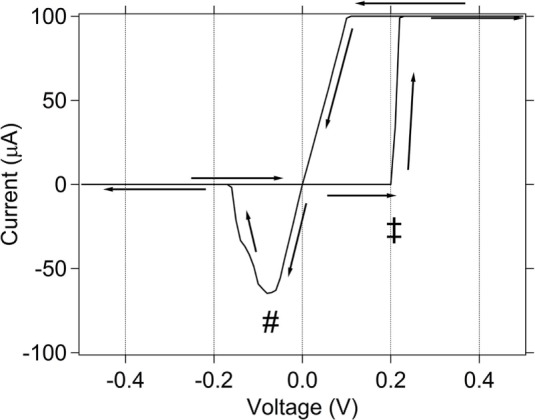
**DC (quasi-static) IV curve for memristor used in this work**. ± denotes the threshold voltage for switching from high to low resistance. # denotes the threshold voltage for switching from low to high resistance. The compliance current used is 100 µA.

A DC (quasi-static) measurement of a typical memristor used in this work exhibits the current–voltage (IV) curve shown in Figure 3. The IV curve shows a device initially in a high resistance state, which is switched to a low resistance with the application of a positive potential and switched to a high resistance through application of a negative potential. To see this in Figure 3, the measurement starts at *V* = 0 and follows the direction of the arrows toward +0.5 V, and then sweeps backwards through *V* = 0 to −0.5 V and back to *V* = 0 V. The transition from a high to low resistance occurs when a threshold voltage is reached (marked with ‡ on IV curve). The transition from low to high resistance occurs at a reverse threshold voltage (marked with # on the IV curve). To prevent the device from being overheated after it switches to a low resistance, a compliance current of 100 µA is used to limit the current. Thus, when the current through the device reaches 100 µA as it switches from high to low resistance, it is clamped at that value. This appears as a flat line in the IV curve at current = 100 µA. This general “bowtie” IV response is characteristic of the SDC device.

**Figure 4:**
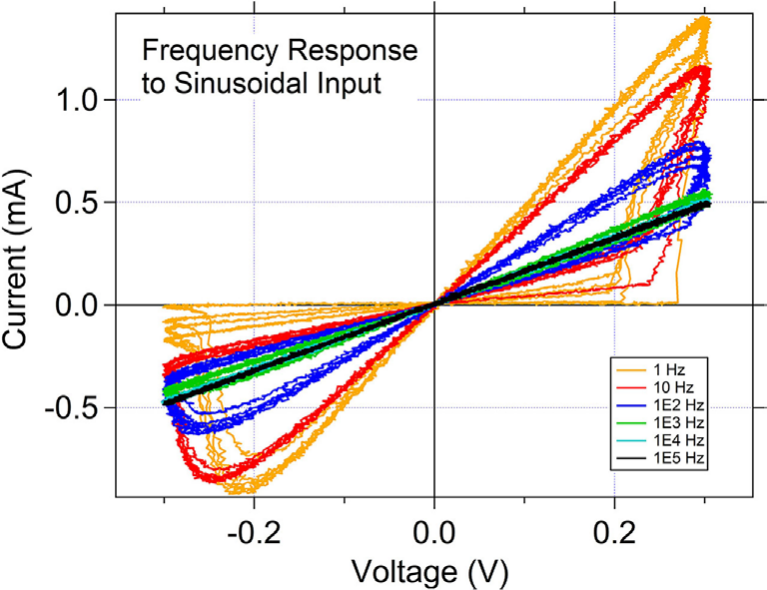
**Response of the memristor to a sinusoidal input signal as a function of frequency**. The response of the memristor to the sinusoid as a function of input signal frequency indicates that this is a generic memristor.

The SDC memristor is classified as a “generic” memristor (Chua, 2015) as shown by the sinudoidal input frequency response IV curves for the memristor (Figure 4). As the frequency of the sinusoidal input is increased from 1 Hz toward 100 kHz, the IV curve shows positive and negative lobes that collapse into a straight line through the IV origin as the frequency is increased. This is one of the hallmark features of the generic memristor. It should be noted clearly that this does not indicate that the frequency response of the device is poor, as is often the misinterpretation when one sees this sinusoidal response. In fact, the device can respond quite well to short (nanoseconds) pulses, as is demonstrated in this and previous (Campbell, 2017) work.

### Electrical Measurements

**Figure 5:**
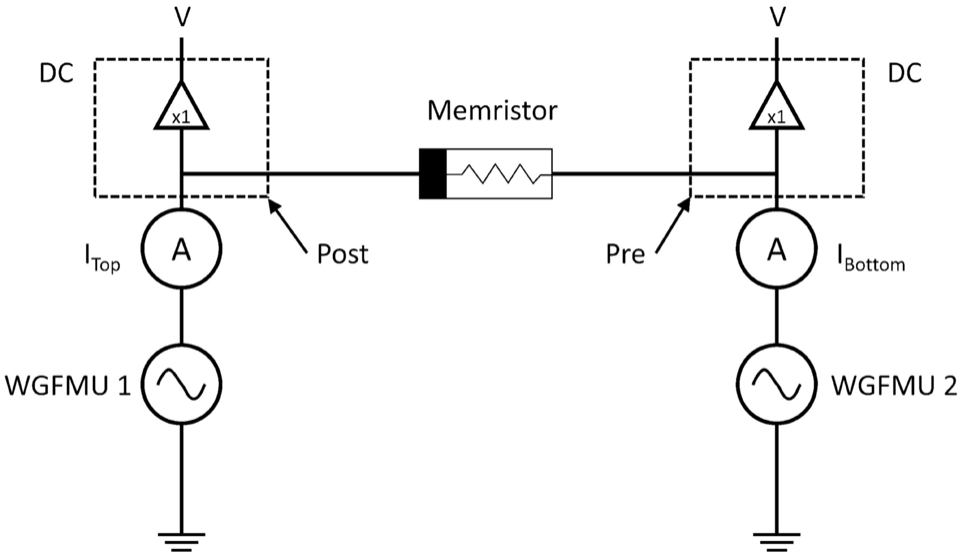
**Electrical measurement test set-up**. The waveform generator/fast measurement units produce the pulses, whereas the RSU channels provide the DC measurement of resistance after every Δ*T* test.

All electrical measurements were performed at the wafer level using a Micromanipulator 6200 microprobe station and an Agilent B1500A Semiconductor Device Parameter Analyzer equipped with two 2-channel waveform generator/fast measurement units (WGFMUs). Each WGFMU was connected to an electrode on the memristor and used to generate the pre- and postsynaptic spikes. Device resistance was read immediately before and after each Δ*T* test by measuring the current during application of 20 mV DC potential across the memristor. A DC potential of 20 mV is low enough to avoid perturbing the state of the device. Figure 5 shows the STDP measurement set-up.

Each spike shape (Figure 2) function was separately created in software using the HP33250A arbitrary waveform generator drawing/creation program (BenchLink Arb) and exported as a waveform file. This function was then included in a C^++^ program, along with the B1500 measurement commands for the STDP tests (described in Section “STDP Experiment”) for each given experiment. This program then controlled the B1500 for the duration of the STDP measurements and then it saved the data from the B1500 in the form of a csv file. Once created, the STDP testing program could be altered for each different pulse shape by simply creating a new spike shape and including that in the command code. Table 1 lists the measurement parameters for the pulse shape tested, the maximum Δ*T* achieved during the test, the time step size for the pulse (resolution between points on the spike), the amplitude of the pulse, and the number of devices tested with those conditions.

**Table 1:** **Spike-timing dependent plasticity Δ*T* timescales and pulse shapes tested**.

Test type	Pulse shape tested	Δ*T* max/step size	Full-width-half-maximum	Amplitude (V)	# Devices tested	Estimated energy requirement per memristor (assuming resistance range of 1 kΩ–1 MΩ)
Long	1	1 s/50 ms	137.5 ms	±0.2	5	0.5 µJ–5 nJ
	2	400/10 ms	104 ms	0.2/−0.1	26	0.4 µJ–0.1 nJ
Med	1	10 ms/500 µs	1.375 ms	±0.35	5	17–0.1 nJ
	2, 3,4	40/1 ms	10.4 ms	0.3/−0.2	10	93–0.1 nJ
Short	1	100/5 µs	13.75 µs	±0.7	5	0.6 nJ–60 pJ
	2	4 µs/100 ns	1.04 µs	0.4/−0.3	10	16–0.9 pJ
Ultra short	1	1 µs/50 ns	137.5 ns	±0.9	10	11–0.1 pJ

### STDP Experiment

In STDP, the difference in time between the firing of pre- and postsynaptic neurons determines whether the “strength” of the connection between those neurons increases or decreases. Biologically, this is caused by a change in the ion concentration in the intracellular space between the neurons making it more or less conductive. It was shown (Bi and Poo, 2001) for a biological synapse that when the presynaptic spike arrives before the postsynaptic spike (Δ*T* > 0), the synapse exhibited increased synaptic weight (Δ*w* > 0) and when the presynaptic spike arrives after the postsynaptic spike (Δ*T* < 0) that the synapse exhibited decreased synaptic weight (Δ*w* < 0). If the two neurons fire close in time, they will have a larger influence on the change in the synapse strength.

**Figure 6:**
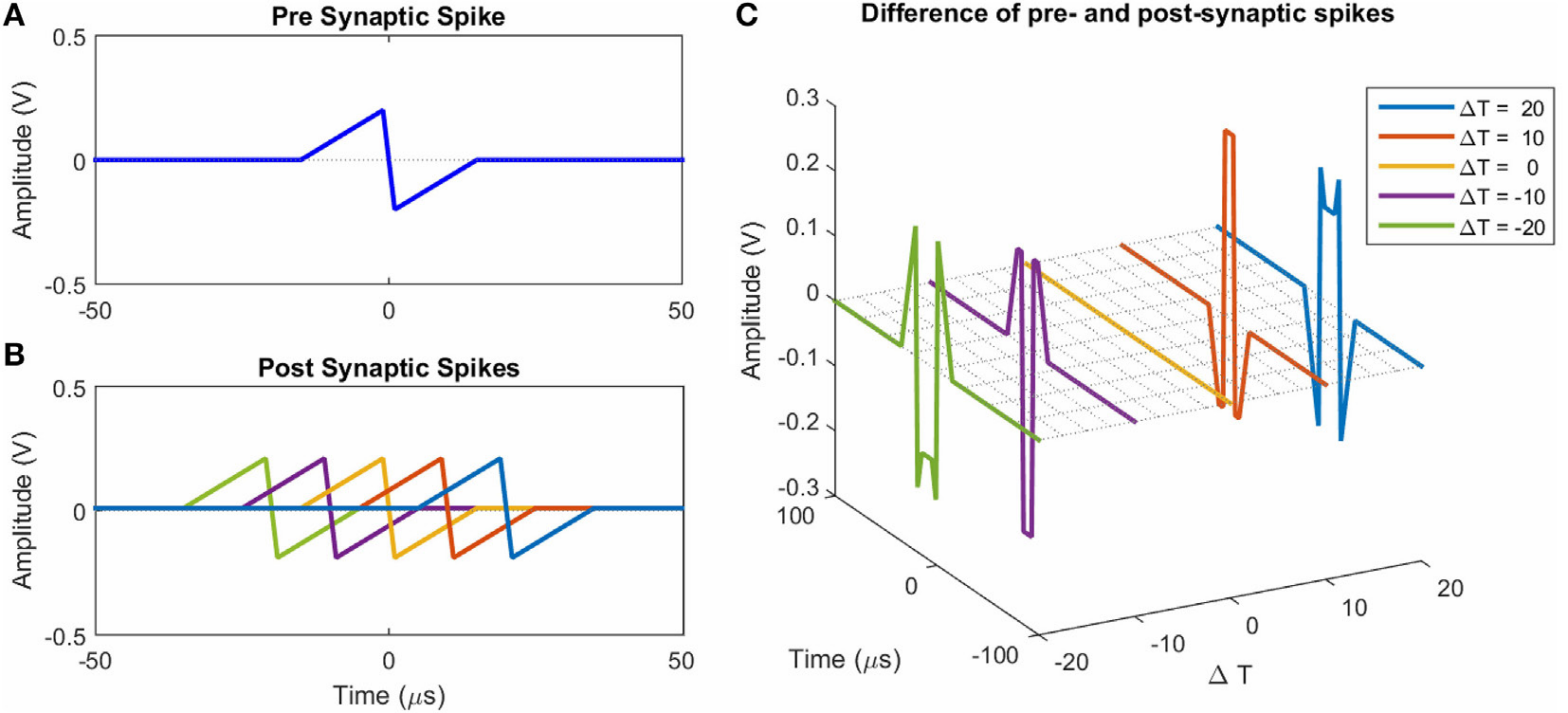
**(A)** An example of a presynaptic spike. **(B)** Examples of postsynaptic spikes. They are at time offsets Δ*T* ∈ {−20, −10, 0, 10, 20} from the presynaptic spike in **(A)**. Here they have the same shape as the presynaptic spike. **(C)** Examples of the net (difference) spike for the five cases in **(B)**.

To illustrate the STDP concept, Figure 6A shows an example of an action potential presynaptic spike function. Five postsynaptic spikes with the same function, just separated in time from the presynaptic spike (Figure 6A) by Δ*T* ∈ {−20, −10, 0, 10, 20}, are shown in Figure 6B. The corresponding difference signals (often referred to as resultant waveforms) between the presynaptic spike and each of the five postsynaptic spikes (plotted against Δ*T* and time) are shown in Figure 6C. Note how the shape, magnitudes, and polarity of the resultant signal changes as a function of Δ*T*. The difference signals are completely dependent on the shapes of the individual spikes. There are many potential pulse shapes that could be used in spiking neural networks that rely on STDP for learning [see examples in Zamarreño-Ramos et al. (2011)]. Four shapes inspired by these were used in the work described here (Figure 2).

In neuromorphic computing, the memristor acts as the synapse and is connected at the two electrode terminals to the pre- and postsynaptic neurons. The STDP experiment emulates nature by applying simple action potentials of the same pulse shape to the two electrodes of the memristor and varying the time between application of these action potentials, Δ*T*, rather than engineering specific pulse shapes in order to achieve desirable resistance programing. The resultant potential waveform across the memristor is the difference between the potentials applied separately to each electrode, resulting in a unique effective programing pulse shape for every Δ*T*. These unique programing pulses will change the resistance of the memristor to an extent that depends on the resultant pulse shape, allowing for a continuous (analog) range of programed resistances over a wide range of pulse widths.

In the STDP experiment performed in this work, a voltage pulse (“spike”) is applied to both electrodes of a memristor synapse at varying times, separated by Δ*T*. The resultant potential difference across the device is the actual programing potential. The STDP synaptic weight change, Δ*w*%, is calculated from the initial (*R*_1_) and final (*R*_2_) resistance:

(1) $$\Delta w\%=\frac{\left(\frac{1}{R_{2}}-\frac{1}{R_{1}}\right)}{\frac{1}{R_{\min}}}\cdot 100$$

where *R*_min_ is the minimum resistance (or maximum conductance) measured for the device over all Δ*T* conditions and is used to normalize the maximum synaptic weight change to 1. The resistances *R*_1_ and *R*_2_ were measured before and after, respectively, a given Δ*T* test. Thus, a decrease in device resistance with application of the STDP pulses corresponds to +Δ*T* in this experiment, and a +Δ*w*%. This means that a pulse is applied to the bottom electrode at a time +Δ*T* before a pulse is applied to the top electrode.

The four spike shapes used in this work (Figure 2) differed by the slope of the rising pulse edge, the slope between negative and positive peaks, and by adding flatness to the positive peak. The tested Δ*T* time ranges spanned from a maximum Δ*T* of 1 s to a minimum of 50 ns. Table 1 provides a summary of the Δ*T* timing used for each pulse shape. Responses on between 5 and 26 devices were measured for each pulse shape and timing test (as provided in Table 1). The results are reported at each Δ*T* measurement showing the average weight% change over the devices tested with the error bar corresponding to the SD of the data set. Error bars extend from ±½ SD. To clarify, if five devices were measured at every Δ*T* for pulse shape 1, then the average of the weight% change at a given Δ*T* (one weight% change at that Δ*T* for each device, corresponding to a total of five values) was calculated from the five values. The SD was calculated from those five values and used to generate the error bar at that Δ*T* point on the STDP graph.

**Figure 7:**
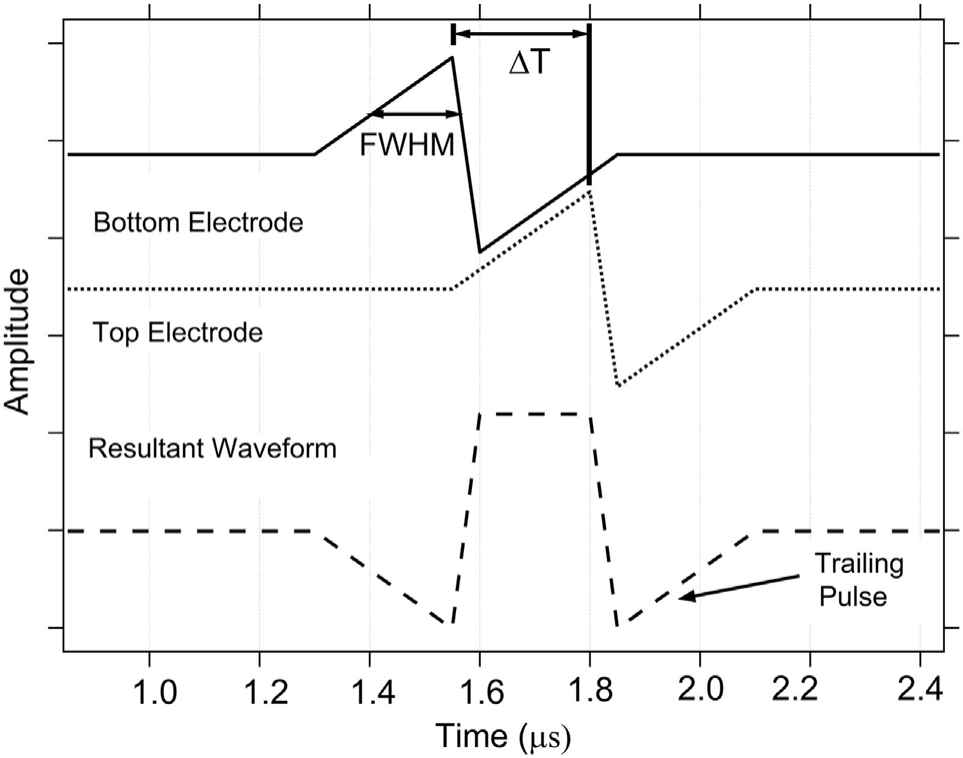
**Example spike-timing dependent plasticity action potential spike pair for shape 1 showing the definitions for Δ*T* and full-width-half-maximum**. Δ*T* defines the time between the peaks of the action potential signals applied to the memristor top and bottom electrodes.

Figure 7 shows an example of a resultant waveform produced by application of spikes with shape 1 applied to the top and bottom electrodes of a memristor, separated in time by Δ*T* = 250 ns. The full-width-half-maximum (FWHM) pulse width is defined on the positive-going portion of each waveform. Δ*T* is defined as the time between the peaks of the positive-going waveform on the top and bottom electrodes. Note that while there is a large positive peak in the center of the resultant waveform, it is followed by a negative peak, labeled trailing pulse, in Figure 7. For the ion-conducting memristors used in this work, the change in resistance is dependent on the electric field induced movement of metal ions within the active device layer. Thus, any portion of the resultant waveform with a potential higher than the minimum value needed move ions within the conductive pathway will change the resistance. Thus, in many Δ*T* cases, this trailing pulse can have a significant impact on the final device state and is, in fact, responsible for achieving an STDP response. An example of the resultant waveform for pulse shape 2 waveforms for the case of four Δ*T*s is given in the Supplementary Material, highlighting the regions that are above the switching voltage threshold for increasing or decreasing the resistance of the device.

The STDP test sequence used for each test type starts with Δ*T* = 0. Δ*T* is then incrementally increased and cycled between −Δ*T* and +Δ*T* (thus cycling between an increase and decrease in resistance) up to the maximum Δ*T*. Between programing steps, the resulting resistance is read. The next programing pulse is then applied to reprogram the memristor. To illustrate, in the Long test (Table 1), a portion of the test sequence from the starting point is: Δ*T* = 0, Read, Δ*T* = −10 ms, Read, Δ*T* = +10 ms, Read, Δ*T* = −20 ms, Read, Δ*T* = +20 ms, and so on, where the Read is the 20 mV DC measurement to determine device resistance. No attempt is made to reset the memristor to an “initial” state before the next programing sequence is begun. The change in resistance is the desired measurement, and when in operation in a network, the effect of the next pulse pair will be on a memristor that is already in an intermediate state.

In addition to the STDP tests, single-sided spike tests were performed where instead of applying the spike potentials to both electrodes, only the resultant potential difference waveform for a given Δ*T* was created, and then applied to the top electrode with the bottom electrode held at ground. The purpose of these tests was to verify the dependence of the resultant potential waveform on device programmability and to compare with the STDP test results at the Δ*T* corresponding to the tested resultant potential waveform. The single-sided tests included a “trailing edge cancelation” test in which the resultant potential amplitude on the last portion of the waveform was reduced (in 25% increments to 100% reduction/cancelation) in order to demonstrate a way to increase the Δ*w*% by a simple pulse modification at a given Δ*T*.

## Results

### STDP Spike Shape and Timescale Tests

#### Time Tests with Pulse Shape 1

**Figure 8:**
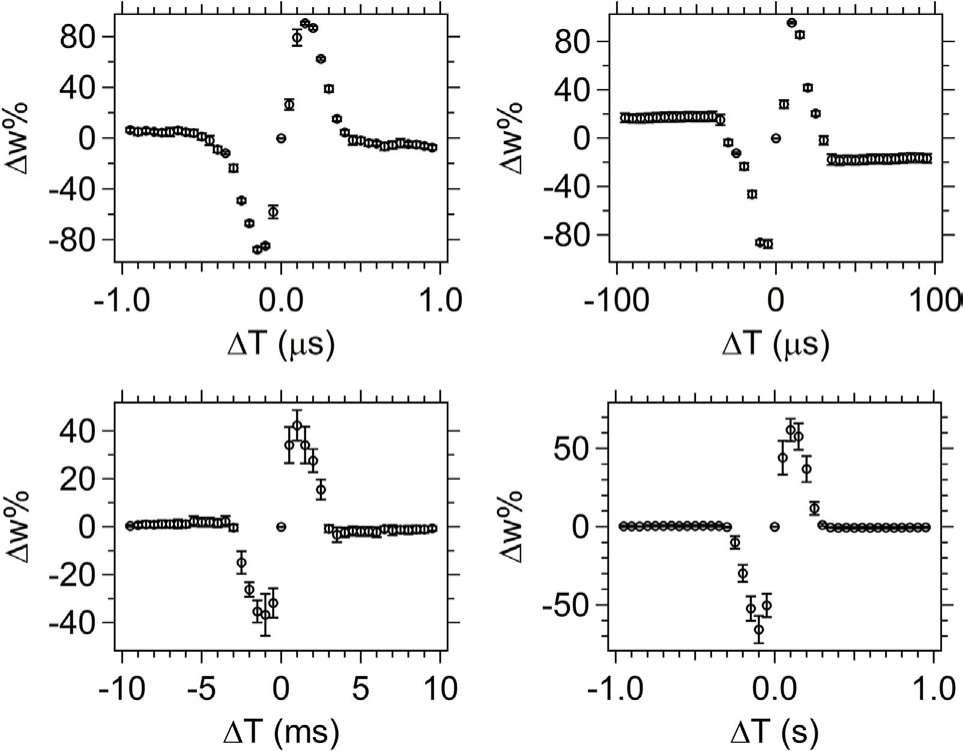
**Spike-timing dependent plasticity results for pulse shape 1 with Δ*T* steps sizes of 50 ns, 5 µs, 500 µs, and 50 ms (starting at top left, clockwise)**. The average weight% change is shown across the set of devices tested (for five different devices, except the 50 ns test which used 10 different devices) with the error bars corresponding to the SD of the data set.

Figure 8 shows average STDP test results for five different devices (except for the 50 ns minimum Δ*T* case which uses 10 devices) using spike pairs with pulse shape 1 over a timescale with a minimum Δ*T* of 50 ns (upper left graph) and a maximum Δ*T* of 1 s (lower right graph). The Δ*T* increment between spike pairs is varied from 50 ns to 50 ms, depending upon the time resolution (Table 1). The overall width of the synaptic weight (Δ*w*%) signal corresponds to the Δ*T* maximum that will still provide overlap between the spikes. Outside of that range, the spikes do not overlap, resulting in a null change in the device resistance.

The STDP results for pulse shape 1 (Figure 8) demonstrate the device not only performs well at the biological timescales (milliseconds range), but has similar performance at the computational timescales (nanoseconds range). This demonstrates the versatility of this memristor for a range of applications, including neuromorphic computing. Error bars on the graph correspond to 1 SD and show good device-to-device repeatability, especially at the shorter programing times. As the programing time increases (bottom two graphs in Figure 8), there is more variation in the device response and the SD is larger. Two possible factors that may contribute to that observation are: (1) increased Joule heating in the device as a function of current through the device for longer times; and (2) increased times allow more mass movement and thus create a larger distribution of resistances due to differing amounts of moved Ag in the device. In general, the devices respond more consistently to shorter pulses when pulse shape 1 is used.

#### Time Tests with Pulse Shape 2

**Figure 9:**
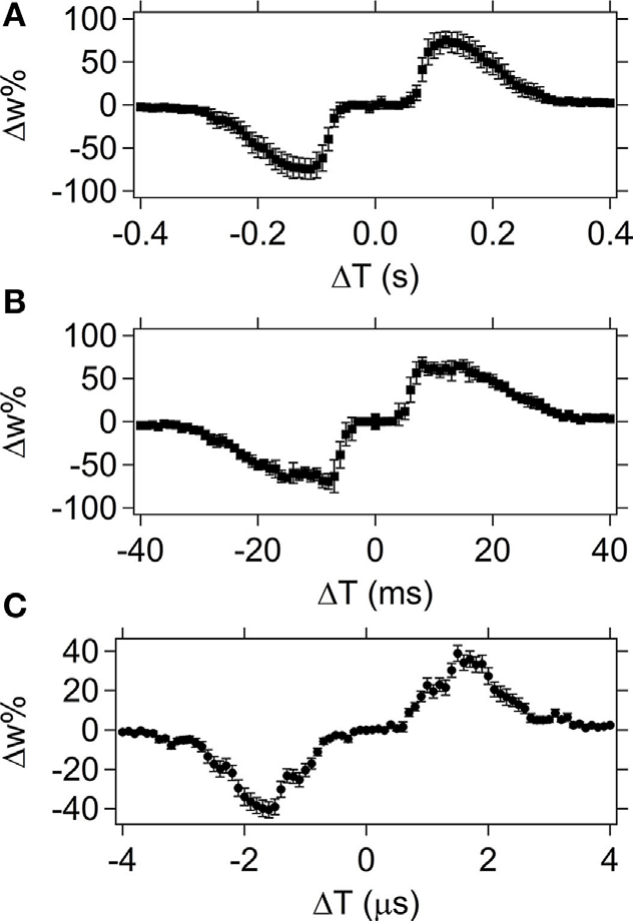
**Spike-timing dependent plasticity results for spike shape 2**. The Δ*T* increment varies from 10 ms to 100 ns in **(A–C)**. The average weight% change is shown across the set of devices tested [26, 10, and 10 different devices **(A–C)**, respectively] with the error bars corresponding to the SD of the data set.

Application of spikes with pulse shape 2 over three different timescales produces the STDP results shown in Figure 9. Overall the Δ*w*% response curve shape is similar at the different time scales. Spike shape 2 has a flat portion on the top of the positive side of the spike, which produces a different global response of the memristor than was seen for pulse shape 1, Figure 8. The flat portion of the spike creates a region around Δ*T* = 0 for which Δ*w*% = 0. This null region is created by the resultant spike difference waveform having a zero potential difference value when the flattened peak on the bottom and top electrode waveforms overlap. This overlap occurs around Δ*T* = 0 and yields a Δ*w*% = 0 segment duration that corresponds to the duration of the flat region on the spike waveform.

The error bars for the shape 2 STDP response are again larger for the longer time measurements (Figure 9A), but in general the Δ*w*% response curves are not as well-defined as in the case of pulse shape 1, which is a consequence of the differences in the resultant waveforms.

Unlike pulse shape 1, if pulse shape 2 was used in a computational setting, the pre- and postsynaptic neurons firing within a short time of each other would not change the synapse behavior. This could be an advantage if one needed to suppress a response; simply modify the spike slightly by “clipping” the pulse positive peak and the output would go to zero for the cases of Δ*T* = duration of the flattened waveform portion.

#### Pulse Shape Tests on the Same Timescale

**Figure 10:**
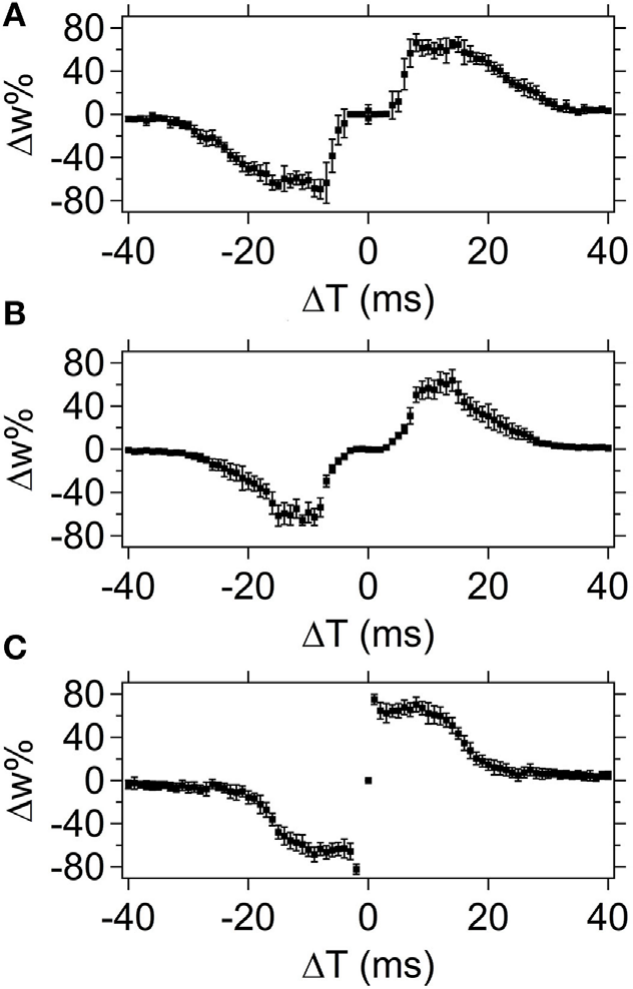
**Comparison of spike-timing dependent plasticity results for spike shapes 2 (A), 3 (B), and 4 (C)**. The average weight% change is shown across the set of devices tested (10 different devices) with the error bars corresponding to the SD of the data set.

Figure 10 compares the STDP Δ*w*% response curves for pulse shapes 2, 3, and 4. Similar to pulse shape 2, pulse shape 3 creates a Δ*w*% = 0 region around Δ*T* = 0 (compare Figures 10A,B). In the case of pulse shape 3, the null region is due to the decreased slope of the transition between the positive and negative peaks compared to the slope that is present in pulse shape 1. This smaller slope again creates a null region in the resultant waveform that corresponds to a Δ*w*% = 0 segment during overlap of the spike pairs.

An increase in the slope between peaks for pulse shape 4, compared to pulse shape 1, causes the null region around Δ*T* = 0 to disappear, and instead a sharp increase in Δ*w*% appears around Δ*T* = 0 compared to the surrounding Δ*T* regions (Figure 10C). The intensity of the sharp increase in Δ*w*% is directly related to the increase in the slope between peaks.

### STDP Single-Sided and Trailing Pulse Suppression Tests with Pulse Shape 1

The resultant pulse for most Δ*T* has a symmetric shape with a positive (negative) peak, a negative (positive) peak, or zone followed by another positive (negative) peak (see Figures 6C and 7). The memristor will experience a state change if any of the peaks in the resultant waveform exceeds the threshold voltage for writing or erasing. These multiple pulses in the resultant contribute ultimately to the overall STDP response, but it is possible to alter the overall STDP response curve by controlling the potential allowed beyond a threshold voltage. It thus might be desirable for the combined system (neurons and synapses) when doing learning and computation to not experience the later reprograming steps. If a pair of pulses could be found that did not produce multiple peaks, the system could have a more desirable global behavior. The effect of the trailing pulse, which is present in the spike pair resultant waveforms for the case of pulse shape 1 (Figure 7), was investigated by choosing one Δ*T* case and applying the resultant waveform to the top electrode, with varying amounts of suppression in the trailing pulse amplitude.

**Figure 11:**
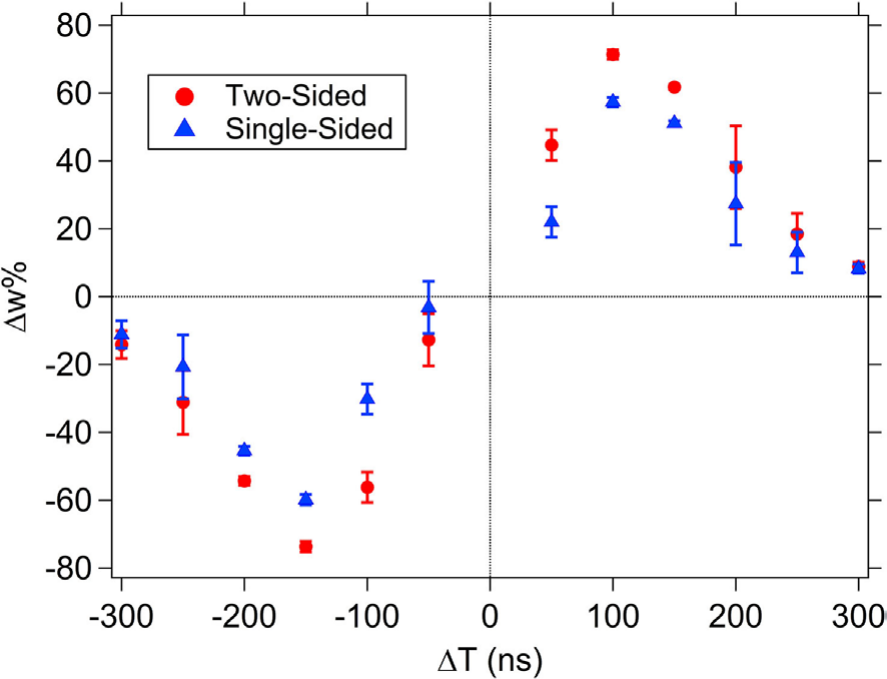
**Spike shape 1 single-sided, resultant-only results compared to the spike-timing dependent plasticity (STDP) results using a spike 1 pair (two-sided)**. The resultant used in each Δ*T* case of the single-sided test is the resultant waveform expected from the STDP test. The average weight% change is shown across the set of devices tested (10 different devices for the two-sided test and 3 for the single-sided test) with the error bars corresponding to the SD of the data set.

To investigate the effect of the resultant pulse shape on memristor programing, first it was verified that applying a signal with the same potential shape as the resultant waveform to the top electrode, while keeping the bottom electrode grounded would have the same overall effect as applying the pre- and postsynaptic pulses to the two terminals. The results of this test, Figure 11, show the expected similarity in shape and approximate Δ*w*% between the normal (two-sided) STDP test with a spike pair and the resultant-only (single-sided) test.

**Figure 12:**
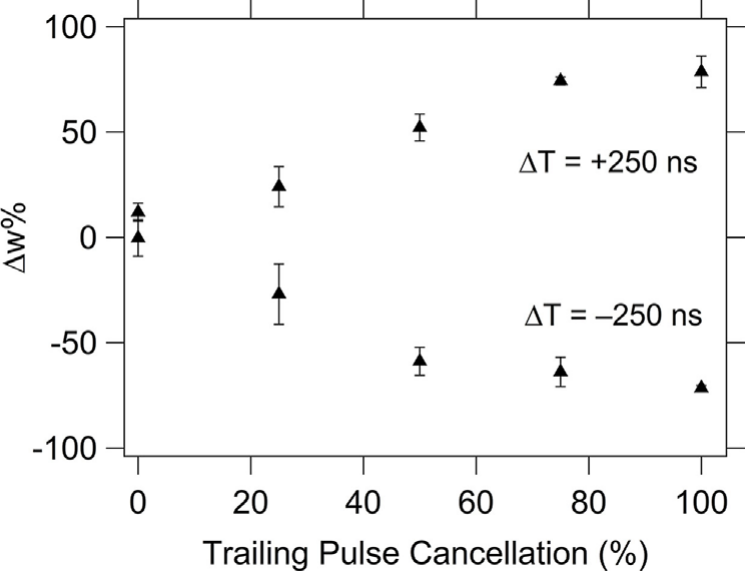
**Effects of reducing the amplitude of the trailing edge pulse on the %Δξ for Δ*T* = ±250 ns**. The average weight% change is shown across the set of devices tested (10 different devices) with the error bars corresponding to the SD of the data set.

To evaluate the effect of changing the trailing pulse amplitude, a fixed value of Δ*T* = ±250 ns was selected based on the data in Figure 11, since they both correspond to regions of low, but non-zero, Δ*w*% so that the effect of altering the amplitude of the trailing pulse will be obvious. For pulse shape 1, the resultant waveform has values that exceed the device’s threshold switching value in both the positive and negative threshold directions (see Figure 7), resulting in changes of resistance both higher and lower values during the application of the pulse. The final portion of the pulse, the “trailing edge”, sets the total Δ*w*%. The full effect of the trailing edge pulse can be seen in Figure 12 where a suppression of 100% (i.e., the last pulse segment is reduced to 0 V) results in a Δ*w*% that is as high as in the maximum Δ*w*% value during the STDP test (Figure 11; this occurs at about Δ*T* = 100 ns). Partial suppression of the trailing edge pulse results in incremental changes to the Δ*w*%.

### Energy Requirements

An estimate of the energy required to change the state of the memristor during STDP requires knowledge of the resistance of the device before, during, and at the conclusion of an applied pulse. Additionally, the resultant waveforms do not have simple pulse shapes. Therefore, an estimate of energy necessary to perform a spike induced change on a memristor is difficult for three major reasons: (1) the device resistance is changing during the application of the resultant waveform, both to higher and lower resistances; (2) the energy per pulse is different for every Δ*T*; and (3) the device resistance can be high initially and change between values of high resistance (lower energy) or it can be low and change between values of low resistance (higher energy).

To get a rough estimate of the energy per memristor, a range was calculated with the worst case assumption that the pulse was a rectangular pulse with an amplitude and pulse width (FWHM) as given in Table 1. It was next assumed that the device resistance was constant in a high resistance range of 100–500 kΩ, or a low resistance range of 1–10 kΩ, both ranges that are reasonable for the SDC device (Campbell, 2017). Using these values, the energy per memristor was estimated and is shown in Table 1. In all cases, these are most likely overestimates of the energy required. However, they do provide a quick look at the potential order of magnitude energy requirements.

## Discussion

Spike-timing dependent plasticity, as developed from Hebbian learning, requires that if the presynaptic spike arrives before the postsynaptic spike that the synapse exhibits long-term potentiation or increased synaptic weight and that if the presynaptic spike arrives after the postsynaptic spike that the synapse exhibits long-term depression or decreased synaptic weight. This effect has been shown for all of the pulse shapes and all the time scales presented in this paper. These change in weight plots do not perfectly match the plots experimentally measured for biologic systems (Bi and Poo, 2001), but since they contain regions of both potentiation and depression, they will be suitable for neuromorphic learning. Through the experiments in this paper, the nature of the potentiation and depression response of this memristor relative to different pulse shapes can be seen.

Neither the null region observed for pulse shapes 2 and 3 nor the spiking region for pulse shape 4, near Δ*T* = 0 was initially expected since the differences between the pulse shapes are subtle. However, the differences are significant enough to impact the STDP results. This “tunability” of the STDP outcome, based on a slight change in the spike pulse, can be both beneficial and detrimental. Since electronics can be prone to noise, stray capacitance and to device mismatch during fabrication, among other possible interferences, it is possible that the spike pulse generated by the circuit could alter the STDP outcome in an undesirable way. Alternatively, one may be able to use this feature to, for example, suppress a circuit output by adding a slight slope or flat peak to the spike. Either way, this result demonstrates the significant influence the pulse shape can have on the STDP outcome and could prove to be a useful feature for circuit designers.

Because there are essentially an infinite number of possible pulse shapes, and the pre- and postsynaptic pulses are not required to be identical in shape, this study provides guidance to allow the design of pulse shape pairs that will have the desired response. For some applications continuing learning (change in synapse resistance) when there is a large Δ*T* is desirable, and the range of Δ*T* to which this continues will depend on the rest of the design components. This was clearly shown in the “trailing edge cancelation” test. In this test, it was shown that a larger Δ*T* is possible by modification of the resultant waveform so that the trailing edge was completely suppressed. In the event that a circuit designer wants to maintain a higher Δ*w*% in a longer Δ*T*, suppression of the trailing edge would provide that opportunity. Additionally, partial suppression of the trailing edge pulse could achieve incremental weight changes if desired.

Compared to a pure CMOS implementation of the STDP learning rule, memristor synapses require significantly less area on a chip than an equivalent CMOS-based synapse and are suitable for use in a cross bar array architecture. For example, a switched-capacitor realization of synapses in 28 nm CMOS was developed (Noack et al., 2015), which minimizes the leakage current problems present when CMOS-based architectures are scaled down in size. However, this system requires 0.36 mm^2^ area and a power consumption of 1.9 mW for only 128 presynapses and 8,192 “stop-learning” synapses which corresponds to roughly 2.27 × 10^4^ synapses/mm^2^ and an energy requirement of 0.23 nJ to 0.23 mJ per synapse. By contrast, 16 Gb ReRAM ion-conducting, chalcogenide-based memory chips have been fabricated at the 27 nm node (Fackenthal et al., 2014) which have a total area of 168 mm^2^, including all periphery circuits required of a memory chip, as well as the memory elements. The memory elements on this chip, CuTe-based ion-conducting devices, are actually memristors, thus providing a good analogy to a high density memristor array. Using this entire chip area, a worst case approximation for synapse density would be at least 9.52 × 10^7^ synapses/mm^2^, or a factor of 1,000 more synapses per mm^2^ than the CMOS-based architecture, even taking into account all of the CMOS circuitry incorporated into the memory chip (periphery circuits and access transistors).

Without knowing the power requirement of the 16 Gb ReRAM chip, it is not possible to know the energy requirement per memristor directly for this chip. However, an estimate of the energy requirements for the memristors used in this work, which are also chalcogenide ion-conductors, is provided in Table 1 for each experiment conducted and is a reasonable approximation to the energy requirements of the memristors in the 16 Gb ReRAM chip given that they are both chalcogenide-based ion-conducting device types. For pulse timing ranging from seconds to nanoseconds and considering the pulse shapes used in this work, the memristor displays lower energy requirements than the CMOS counterpart. In fact, as the memristor is driven with faster pulses, the overall energy requirement decreases even more (nanojoules to picojoules per memristor). From this, it can easily be seen that two major advantages of the memristor in the STDP application are: (1) denser area achievable and (2) lower energy requirements. In fact, from the energy requirements, it is clear that one of the advantages of operating a memristor on the faster timescale (nanoseconds), even though it is not a biological timescale, is that artificial synapses are not limited to slow, biological speeds and can therefore take advantage of the greatly reduced energy requirements of the memristors at fast speeds.

Even though to date there has been no report of large scale integration of memristors as synapses, it is promising that there has been demonstrated a large scale memory chip which uses memristors (Fackenthal et al., 2014). While this report focused on the ion-conducting CuTe device as a binary memory, this device is a memristor and thus the memory chip does demonstrate the large scale feasibility of memristor incorporation into a large scale CMOS-based integrated circuit. Given this example, the major challenges of integration of new memristor device technologies into large scale integrated circuits can be overcome. These challenges include how to repeatably achieve novel material deposition for device-to-device and lot-to-lot consistency, as well as fundamental issues with trying to access a variable resistance device with transistors which, in the traditional architecture with the memristor in the source of the transistor, do not allow a constant switching voltage across a device due to a voltage divider between the access transistor ON resistance and the memristor.

## Conclusion

Ge_2_Se_3_/SnSe/Ag-based ion-conducting memristive devices perform over the seconds to nanoseconds timescale as synapses in an STDP experiment. STDP tests were performed with four different spike wave shapes in order to demonstrate the influence of the resultant waveforms on the STDP response. This wave shape analysis can be used to help choose the pulse shape to be used in future circuit designs. Furthermore, the ability of the memristor to operate on a large dynamic range timescale, allows for one memristor type to be used for applications requiring short or long timescales. This provides an opportunity for a single integrated circuit to include both long and short timescale applications and to have only one type of memristor that needs to be integrated with the circuit during fabrication. Without this possibility, it would be unlikely that multiple memristor types which catered to different timescales could be fabricated together on one integrated circuit.

The data retention measurements, or lifetime of a particular resistance state after the application of a spike pair, are currently in progress.

## Author Contributions

KC designed and fabricated the memristor devices, designed the STDP experiments, analyzed the data, and wrote the manuscript; KD performed measurements, wrote instrument control and data collection programs, and analyzed data; ES analyzed data, performed pulse shape simulations, and assisted in writing the manuscript.

## Conflict of Interest Statement

The authors declare that the research was conducted in the absence of any commercial or financial relationships that could be construed as a potential conflict of interest.

## References

[B1] Alibart F. Pleutin S. Bichler O. Gamrat C. Serrano-Gotarredona T. Linares-Barranco B. (2012). A memristive nanoparticle/organic hybrid synapstor for neuroinspired computing. Adv. Funct. Mater. 22, 609–616. 10.1002/adfm.201101935

[B2] Banerjee S. Chakravorty D. (1999). Electrical resistivity of silver-silica nanocomposites. J. Appl. Phys. 85, 3623–3625. 10.1063/1.369724

[B3] Bi G. Poo M. M. (2001). Synaptic modification by correlated activity: Hebb’s postulate revisited. Ann. Rev. Neurosci. 24, 139–166. 10.1146/annurev.neuro.24.1.139 11283308

[B4] Cabaret T. Fillaud L. Jousselme B. Klein J.-O. Derycke V. (2014). “Electro-grafted organic memristors: properties and prospects for artificial neural networks based on STDP,” in Proceedings of the 14th IEEE International Conference on Nanotechnology, Toronto, CA, 499–504.

[B5] Campbell K. A. (2008a). Method of Forming a PCRAM Device Incorporating a Resistance-Variable Chalcogenide Element. US Patent No. 7,354,793. Washington, DC: U.S. Patent and Trademark Office.

[B6] Campbell K. A. (2008b). Resistance Variable Memory Device and Method of Fabrication. US Patent No. 7,348,209. Washington, DC: U.S. Patent and Trademark Office.

[B7] Campbell K. A. (2017). Self-directed channel memristor for high temperature operation. Microelectron. J. 59C, 10–14. 10.1016/j.mejo.2016.11.006

[B8] Campbell K. A. Anderson C. M. (2007). Phase-change memory devices with stacked Ge-chalcogenide/Sn-chalcogenide layers. Microelectron. J. 38, 52–59. 10.1016/j.mejo.2006.09.012

[B9] Chang T. Jo S.-H. Kim K.-H. Sheridan P. Gaba S. Lu W. (2011). Synaptic behaviors and modeling of a metal oxide memristive device. Appl. Phys A. 102, 857–863. 10.1007/s00339-011-6296-1

[B10] Chua L. (2015). Everything you wish to know about memristors but are afraid to ask. Radioengineering 24, 331–368. 10.13164/re.2015.0319

[B11] Cruz-Albrecht J. M. Yung M. W. Srinivasa N. (2012). Energy-efficient neuron, synapse and STDP integrated circuits. IEEE Trans. Biomed. Circuits Syst. 6, 246–256. 10.1109/TBCAS.2011.2174152 23853146

[B12] Dayan P. Abbott L. F. (2001). Theoretical Neuroscience, 1st Edn Cambridge MA: The MIT Press.

[B13] Devasia A. Kurinec S. Campbell K. A. Raoux S. (2010). Influence of Sn migration on phase transition in GeTe and Ge_2_Se_3_ thin films. Appl. Phys. Lett. 96, 141908/1–141908/3. 10.1063/1.3385781

[B14] Devasia A. MacMahon D. Raoux S. Campbell K. A. Kurinec S. K. (2012). Investigation of inter-diffusion in bilayer GeTe/SnSe phase change memory films. Thin Solid Films 520, 3931–3935. 10.1016/j.tsf.2012.02.005

[B15] Erokhin V. Fontana M. P. (2011). Thin film electrochemical memristive systems for bio-inspired computation. J. Comput. Theor. Nanosci. 8, 313–330. 10.1166/jctn.2011.1695

[B16] Fackenthal R. Kitagawa M. Otsuka W. Prall K. Mills D. Tsutsui K. (2014). “A 16Gb ReRAM with 200MB/s write and 1GB/s read in 27nm technology,” in Solid-State Circuits Conference Digest of Technical Papers (ISSCC), 2014 IEEE International, San Francisco, CA, 338–340. 10.1109/ISSCC.2014.6757460

[B17] Gaba S. Sheridan P. Zhou J. Choi S. Lu W. (2013). Stochastic memristive devices for computing and neuromorphic applications. Nanoscale 5, 5872–5878. 10.1039/c3nr01176c 23698627

[B18] Ielmini D. Waser R. (eds) (2016). Resistive Switching. New York, NY: Wiley-VCH.

[B19] Jo S. H. Chang T. Ebong I. Bhadviya B. B. Mazumder P. Lu W. (2010). Nanoscale memristor device as synapse in neuromorphic systems. Nano Lett. 10, 1297–1301. 10.1021/nl904092h 20192230

[B20] Kamalanathan D. Russo U. Ielmini D. Kozicki M. N. (2009). Voltage-driven on-off transition and tradeoff with program and erase current in programmable metallization cell (PMC) memory. IEEE Electron Dev. Lett. 30, 553–555. 10.1109/LED.2009.2016991

[B21] Kim S. Du C. Sheridan P. Ma W. Choi S. Lu W. D. (2015). Experimental demonstration of a second-order memristor and its ability to biorealistically implement synaptic plasticity. Nano Lett. 15, 2203–2211. 10.1021/acs.nanolett.5b00697 25710872

[B22] Kozicki M. N. Mitkova M. (2006). Mass transport in chalcogenide electrolyte films – materials and applications. J. Non Cryst. Solids. 352, 567–577. 10.1016/j.jnoncrysol.2005.11.065

[B23] Krzysteczko P. Münchenberger J. Schäfers M. Reiss G. Thomas A. (2012). The memristive magnetic tunnel junction as a nanoscopic synapse-neuron system. Adv. Mater. 24, 762–766. 10.1002/adma.201103723 22223304

[B24] La Barbera S. Vuillaume D. Alibart F. (2015). Filamentary switching: synaptic plasticity through device volatility. ACS Nano 9, 941–949. 10.1021/nn506735m 25581249

[B25] Li S. Zeng F. Chen C. Liu H. Tang G. Gao S. (2013a). Synaptic plasticity and learning behaviours mimicked through Ag interface movement in an Ag/conducting polymer/Ta memristive system. J. Mater. Chem. C 1, 5292–5298. 10.1039/c3tc30575a

[B26] Li Y. Zhong Y. Xu L. Zhang J. Xu X. Sun H. (2013b). Ultrafast synaptic events in a chalcogenide memristor. Sci. Rep. 3, 1619. 10.1038/srep01619 23563810 PMC3619133

[B27] Luo W. Yuan F.-Y. Wu H. Pan L. Deng N. Zeng F. (2015). “Synaptic learning behaviors achieved by metal ion migration in a Cu/PEDOT:PSS/Ta memristor,” in IEEE 15th Non-Volatile Memory Technology Symposium (NVMTS) China: Tsinghua University at Beijing10.1109/NVMTS.2015.7457490

[B28] Mahalanabis D. Barnaby H. J. Gonzalez-Velo Y. Kozicki M. N. Vrudhula S. Dandamudi P. (2014a). Incremental resistance programming of programmable metallization cells for use as electronic synapses. Solid State Electron. 100, 39–44. 10.1016/j.sse.2014.07.002

[B29] Mahalanabis D. Gonzalez-Velo Y. Barnaby H. J. Kozicki M. N. Dandamudi P. Vrudhula S. (2014b). Impedance measurement and characterization of Ag-Ge30Se70-based programmable metallization cells. IEEE Trans. Electron Dev. 61, 3723–3730. 10.1109/TED.2014.2358573

[B30] Mahalanabis D. Sivaraj M. Chen W. Shah S. Barnaby H. (2016). “Demonstration of spike timing dependent plasticity in CBRAM devices with silicon neurons,” in IEEE Int’l Symposium on Circuits and Systems (ISCAS) Montreal, Canada 10.1109/ISCAS.2016.7539047

[B31] Mandal S. El-Amin A. Alexander K. Rajendran B. Jha R. (2014). Novel synaptic memory device for neuromorphic computing. Sci. Rep. 4, 5333. 10.1038/srep05333 24939247 PMC4061545

[B32] Mitkova M. Kozicki M. N. (2002). Silver incorporation in Ge-Se glasses used in programmable metallization cell devices. J. Non Cryst. Solids. 299–302(pt. B), 1023–1027. 10.1016/S0022-3093(01)01068-7

[B33] Noack M. Partzsch J. Mayr C. G. Hänzsche S. Scholze S. Höppner S. (2015). Switched-capacitor realization of presynaptic short-term-plasticity and stop-learning synapses in 28 nm CMOS. Front. Neurosci. 9:10. 10.3389/fnins.2015.00010 25698914 PMC4313588

[B34] Pickett M. D. Medeiros-Ribeiro G. Williams R. S. (2013). A scalable neuristor built with Mott memristors. Nat. Mater. 12, 114–117. 10.1038/nmat3510 23241533

[B35] Qu B. Younis A. Chu D. (2016). Recent progress in tungsten oxides based memristors and their neuromphological applications. Electron. Mater. Lett. 12, 715 10.1007/s13391-016-6129-7

[B36] Rachmuth G. Shouval H. Z. Bear M. F. Poon C.-S. (2011). A biophysically-based neuromorphic model of spike rate- and timing-dependent plasticity. PNAS 108, E1266–E1274. 10.1073/pnas.1106161108 22089232 PMC3241759

[B37] Rajabi S. Saremi M. Barnaby H. J. Edwards A. Kozicki M. N. Mitkova M. (2015). Static impedance behavior of programmable metallization cells. Solid State Electron. 106, 27–33. 10.1016/j.sse.2014.12.019

[B38] Rajendran B. Liu Y. Seo J.-S. Gopalakrishnan K. Chang L. Friedman D. J. (2013). Specifications of nanoscale devices and circuits for neuromorphic computational systems. IEEE Trans. Electron Dev. 60, 246–253. 10.1109/TED.2012.2227969

[B39] Regner J. Balasubramanian M. Cook B. Li Y. Kassayebetre H. Sharma A. (2009). “Integration of IC industry feature sizes with university back-end-of-line post processing: example using a phase-change memory test chip,” in Microelectronics and Electron Devices, 2009. WMED 2009. IEEE Workshop on, Boise, ID 10.1109/WMED.2009.4816141

[B40] Rose G. S. Pino R. Wu Q. (2011a). “Exploiting memristance for low-energy neuromorphic computing hardware,” in Circuits and Systems (ISCAS), 2011 IEEE International Symposium on, Rio de Janeiro, 2942–2945. 10.1109/ISCAS.2011.5938208

[B41] Rose G. S. Pino R. Wu Q. (2011b). “A low-power memristive neuromorphic circuit utilizing a global/local training mechanism,” in Neural Networks (IJCNN), The 2011 International Joint Conference on, San Jose, CA, Brazil, 2080–2086. 10.1109/IJCNN.2011.6033483

[B42] Serrano-Gotarredona T. Masquelier T. Prodromakis T. Indiveri G. Linares-Barranco B. (2013a). STDP and STDP variations with memristors for spiking neuromorphic learning systems. Front. Neurosci. 7:2 10.3389/fnins.2013.00002 23423540 PMC3575074

[B43] Serrano-Gotarredona T. Prodromakis T. Linares-Barranco B. (2013b). A proposal for Hybrid memristor-CMOS spiking neuromorphic learning systems. IEEE Circuits Syst. Mag. 2013, 74–88. 10.1109/MCAS.2013.2256271 PMC3575074 23423540

[B44] Snider G. Amerson R. Carter D. Abdalla H. Qureshi M. S. Léveillé J. (2011). From synapses to circuitry using memristive memory to explore the electronic brain. IEEE Comput. Mag. 44, 21–28. 10.1109/MC.2011.48

[B45] Subramaniam A. Cantley K. D. Bersuker G. Gilmer D. C. Vogel E. M. (2013). Spike-timing-dependent plasticity using biologically realistic action potentials and low-temperature materials. IEEE Trans. Nanotechol. 12, 450–459. 10.1109/TNANO.2013.2256366

[B46] Thomas A. (2013). Memristor-based neural networks. J. Phys. D Appl. Phys. 46, 093001 10.1088/0022-3727/46/9/093001

[B47] Wang F. Dunn W. P. Jain M. De Leo C. Vickers N. (2011). The effects of active layer thickness on programmable metallization cell based on Ag-Ge-S. Solid State Electron. 61, 33–37. 10.1016/j.sse.2011.01.042

[B48] Wang H. Li H. Pino R. E. (2012a). “Memristor-based synapse design and training scheme for neuromorphic computing architecture,” in Neural Networks (IJCNN), The 2012 International Joint Conference on, Brisbane, Austrailia 10.1109/IJCNN.2012.6252577

[B49] Wang Z. Q. Xu H. Y. Li X. H. Yu H. Liu Y. C. Zhu X. J. (2012b). Synaptic learning and memory functions achieved using oxygen ion migration/diffusion in an amorphous InGaZnO memristor. Adv. Funct. Mater. 22, 2759–2765. 10.1002/adfm.201290076

[B50] Wang Z. Minghui Y. Zhang T. Cai Y. Want Y. Yang Y. (2016). Engineering incremental resistive switching in TaO_x_ based memristors for brain-inspired computing. Nanoscale 8, 14015–14022. 10.1039/c6nr00476h 27143476

[B51] Waser R. Dittmann R. Staikov G. Szot K. (2009). Redox-based resistive switching memories – nanoionic mechanisms, prospects, and challenges. Adv. Mat. 21, 2632–2663. 10.1002/adma.200900375 36751064

[B52] Wu Y. Yu S. Wong H.-S. P. Chen Y.-S. Lee H.-Y. Wang S.-M. (2012). “AlOx-based resistive switching device with gradual resistance modulation for neuromorphic device application,” in 4th IEEE International Memory Workshop Milano, Italy 10.1109/IMW.2012.6213663

[B53] Yu S. Wu Y. Jeyasingh R. Kuzum D. Wong H.-S. P. (2011). An electronic synapse device based on metal oxide resistive switching memory for neuromorophic computation. IEEE Trans. Electron Dev. 58, 2729–2737. 10.1109/TED.2011.2147791

[B54] Zamarreño-Ramos C. Camuñas-Mesa L. A. Pérez-Carrasco J. A. Masquelier T. Serrano-Gotarredona T. Linares-Barranco B. (2011). On spike-timing-dependent-plasticity, memristive devices, and building a self-learning visual cortex. Front. Neurosci. 5:26. 10.3389/fnins.2011.00026 21442012 PMC3062969

[B55] Zhu L. Q. Wan C. J. Guo L. Q. Shi Y. Wan Q. (2014). Artificial synapse network on inorganic proton conductor for neuromorphic systems. Nat. Commun. 5, 3158. 10.1038/ncomms4158 24452193

