# Supplementary figures and images for: Pulse Shape and Timing Dependence on the Spike-Timing Dependent Plasticity Response of Ion-Conducting Memristors as Synapses
Source: Front Bioeng Biotechnol. 2016 Dec 26;4:97. doi: 10.3389/fbioe.2016.00097 (PMC5183647; doi:10.3389/fbioe.2016.00097)

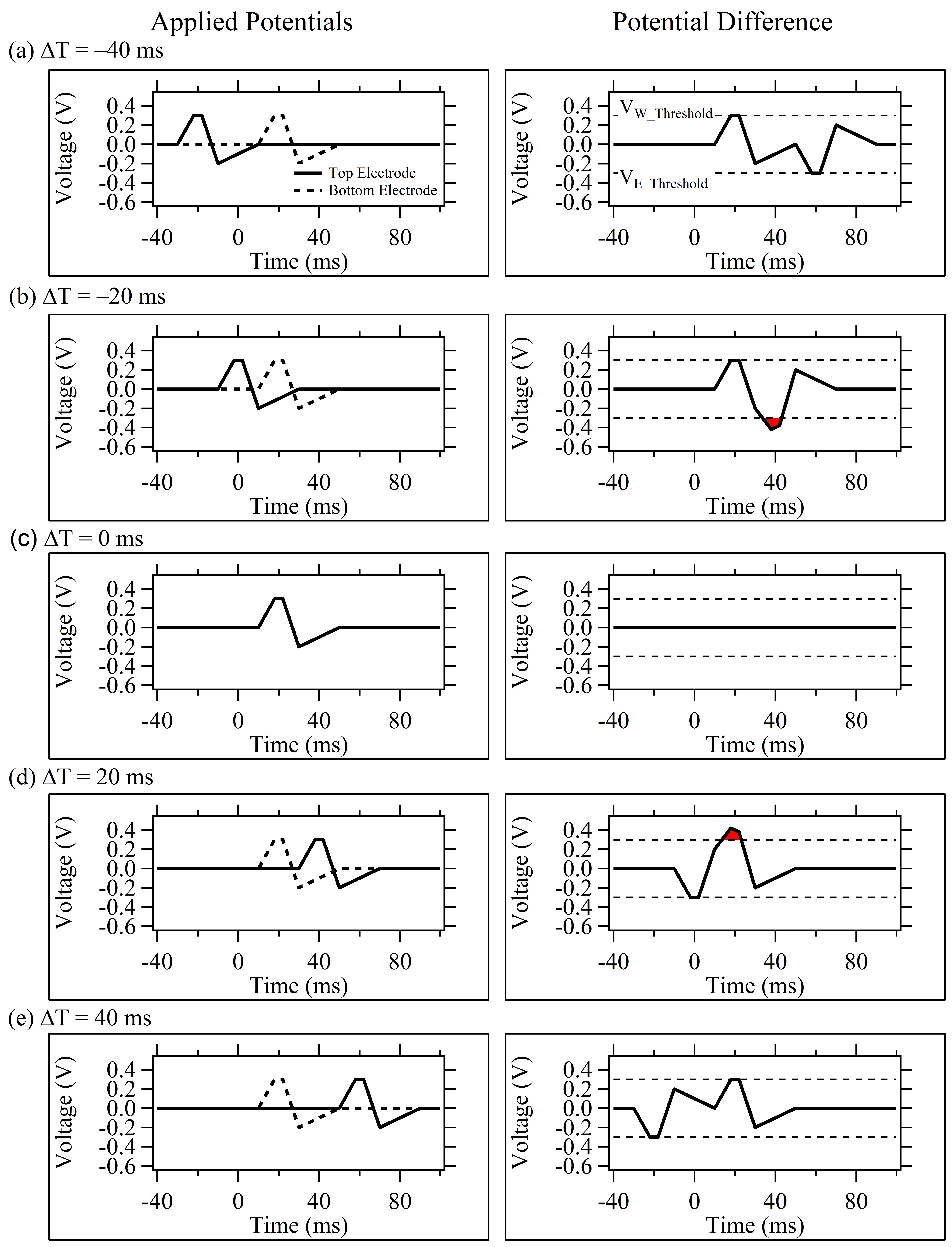

Supplement: Supplementary file 1 [file Image_1.JPEG]
